# The Person Beneath the Hair: Hair Discrimination, Health, and Well-Being

**DOI:** 10.1089/heq.2022.0118

**Published:** 2023-08-02

**Authors:** Manka Nkimbeng, Bernice B. Malaika Rumala, Crystal M. Richardson, Shemekka Ebony Stewart-Isaacs, Janiece L. Taylor

**Affiliations:** ^1^ School of Public Health, University of Minnesota, Minneapolis, Minnesota, USA.; ^2^ CROWNCampaign.com, California, USA.; ^3^ People with Lived Experience Institute, California, USA.; ^4^ The Law Office of Crystal M. Richardson PLLC, High Point, North Carolina, USA.; ^5^ I Am Brilliant, Raleigh, North Carolina, USA.; ^6^ Johns Hopkins School of Nursing, Baltimore, Maryland, USA.

**Keywords:** hair bias, hair discrimination, racial discrimination, black hair, racism, natural hair

## Abstract

Discrimination toward black hair is pervasive in today's society. Hair discrimination is negative bias manifested toward black natural or textured hair styles typically worn by persons of African descent. This commentary discusses the potential effects of hair discrimination on the health and well-being of persons of African descent. Specifically, it explores the mental and physical health implications of hair discrimination and situates it within the broader context of social determinants of health. The Creating a Respectful and Open World for Natural Hair Act has been recently passed in the United States House of Representatives, but more work is needed to eliminate hair discrimination and its negative effects.

## Introduction

Hair discrimination that is sometimes referred to as hair bias, hair racism, or hair harassment is defined as negative stereotypes and attitudes manifested toward natural or black textured hair styles (hereto after referred to only as natural hair).^1^ Natural hair describes texture that is tightly coiled and/or styles that are typically worn by Black persons including afros, locs, twist-outs, and braids.^2^ Society's view of natural hair as unattractive and unmanageable was prevalent during slavery, wherein slave owners required Black women to cover their hair or adopt grooming practices that emulated White/Eurocentric beauty standards.^3^

Hair discrimination is a form of racial discrimination, which is defined as differential treatment based on race or other inadequately justified factors that disadvantages a racial/ethnic group.^4^ Racial discrimination is associated with numerous poor health outcomes.

The surge in media coverage, societal conversation, and policy change is growing evidence for the impact of hair discrimination. Media stories of hair discrimination are reported in schools (from kindergarten to universities), at work (the military, public office, etc.), and within inter-/intrapersonal relationships.^5^ For example, a 2022 New York Times piece describes five kids' experiences of hair discrimination in their various schools.^6^

This commentary explores the potential effects of hair discrimination on the health and well-being of people of African descent. Specifically, it discusses the mental and physical health implications of hair discrimination. These are discussed within the broader context of social determinants of health and health inequities.

### A note about our terminology

Although people of all races/ethnicity have and wear their hair in its natural form, often, the term natural hair in national discourse, especially in Western countries, is frequently related to the textured hair and hair styles worn by Black persons and other people of African descent. The term Black or people of African descent in this article refers to anyone who has their origins from the Black racial groups of Africa.^7^

## Natural Hair Trajectory

Beginning in Africa, hair was a cultural marker of civilization and it was used to indicate wealth, rank, and tribal affiliation.^8,9^ In the United States, enslaved Africans with natural hair worked in the fields, whereas those who worked inside the home wore their hair in patterns similar to the slave masters' straight hair.^10^ These were the origins of straightening natural hair, which has been prejudicially described as “good hair.^10^ In contrast, natural hair without any treatment is referred to as “bad hair” or “unprofessional.”^2^

This inferior view of natural hair was deeply tied to slavery and its depiction of persons of African descent in the United States. To challenge this narrative, natural hair was used as a symbol of power and identity in political discourse in the 70s and 80s Black Power Movement.^11^ The Black Power Movement discouraged its followers against hair straightening and encouraged Afrocentric styles.^11,12^

The continuous growth of Black identity gave birth to the natural hair movement that pushed for greater acceptance of natural hair/hairstyles in the early 2000s.^2^ Many persons of African descent began transitioning from hair straightening to wearing their hair in its natural form.^2,13,14^ Attitudes toward natural hair in today's society are changing but continue to be mixed. For example, Johnson et al. found that compared with Blacks, Whites had more negative attitudes toward natural hair.^1^ In addition, personal narratives and media stories still show some negative attitudes and prejudices toward natural hair or persons transitioning to natural hair, from their families, at school, or in the workplace.^16^

Schools, employers, and other organizational systems have grooming policies and requirements that also influence hairstyles for persons with natural hair. For example, in 2014, the military grooming policy banned several hairstyles including twists and locs but it has since changed this policy.^16^ Some employers have banned certain hairstyles outright and/or require hairstyles that extend to a certain number of inches of hair from the scalp, thereby also limiting certain styles (e.g., Afros).^3^ Many school dress and appearance policies/codes have also been described as discriminatory.^6,17^ Such policies interact with societal attitudes and narratives to exert influence on an individual's hairstyle choice.^18^

## Natural Hair and Mental Health

Hair, beauty, self-image, and identity are inextricably linked and influence each other. Hair and hairstyles have been reported as an important aspect of beauty, confidence, and self-identity.^11,19^ Hairstyle choice is a response to numerous factors including individual perception of beauty and its relation to dominant beauty standards. Therefore, those who believe straightened hair is “good hair” or beautiful may choose to change their natural hair in pursuit of this beauty ideal.^10^ In contrast, some persons may decide to style their hair against their natural preferences due to “fear of” or to prevent hair discrimination.

Black women report being frustrated at the amount of time and effort it takes to hide salient racialized characteristics about themselves including their hair to “fit in” for job interviews or in new job situations.^20^ Hair discrimination can constrain individual choice and impact self-confidence and self-identity.

Hair discrimination may have significant effects on self-image, health, and well-being. Discrimination is described a stressor that activates and prolongs the stress response system.^9,21^ The internalization of negative prejudices can also lead to heightened and prolonged stress responses.^21^ Some people may internalize the negative stereotypes of natural hair including “nappy,” “kinky,” “unprofessional,” or “bad hair,” whereas straightened hair is “good hair” that may ultimately affect the perceptions of beauty and stress. Discrimination is associated with several mental health outcomes (e.g., changes in self-esteem, self-identity, and anxiety).^9,21,22^

Hair discrimination in school-age children is perpetuated through bullying, and school appearance policies and codes. Also called hair-bullying, there are a plethora of stories in the media of children who have been bullied by their peers and teachers.^23–26^ Negative effects of bullying in school-age children include poor academic achievement and absenteeism.^27^ Bullying is also associated with poor mental health outcomes including depression, anxiety, self-harm, and suicidality.^27^ The effects of childhood bullying not only impact current health but can also persist well into adulthood.

In a letter of support for antihair discrimination laws across the country, the Association of Black Psychologists called hair discrimination an “esthetic trauma” and note that the mental health effects of hair discrimination are dire.^15^ In calling it a trauma with dire mental health effects, these professionals have elevated hair discrimination to parallel other known traumas and traumatic events whose negative health effects are well known and studied.

Therefore, hair discrimination invariably affects emotional and mental health and has led to development of coping strategies that include the creation of blogs to support Black women in their natural hair journeys.^28,29^ In their study of 40 blogs, Davis et al. note that blogs have been created with the clear intention of offering informal support to other persons with natural hair, with one blog being described as “hair therapy.”^28^

## (Un)Natural Hair and Physical Health

There are many grooming products and styles used to straighten and maintain tightly coiled and curled natural hair.^3^ Some of these products and processes are harmful or have been associated with negative effects. Localized effects of heat and chemical treatments include burns, scarring, temporary, and permanent hair loss.^30^ Studies show that the use of chemicals for hair care might be associated with allergic reactions, cancer, and fibroids that can affect fertility and increased need for high-risk surgical interventions including hysterectomies.^3,31^

Despite these side effects, we note that everyone should have the choice to style their hair as they chose and should not be required to succumb to biased hair standards. Also, there is a need to ensure that harmful products are removed from the market place so that persons who want to straighten their natural hair can do so safely.

In addition, because it is costly and time consuming to straighten natural hair, some women have reported the avoidance of physical activity and exercise due to the effects that this can have on straight hair styles.^5^ For example, in a sample of 50 African American girls, 24% reported that their hairstyle affected gym participation, and that sweating in gym class will mess-up the hairstyles.^9^ More money and time spent on hair maintenance in this study was associated with decreased physical activity.^9^ Abstaining from physical activity impacts preventive health behaviors that are related to the prevention of chronic diseases such as obesity and diabetes: conditions that disproportionately affect minoritized persons and communities.

Costs and time spent on hair or using a significant portion of income for potentially unnecessary hair maintenance can affect other lifestyle choices. Also, several instances of hair discrimination have resulted in job loss or not acquiring a job.^32^ For example, in the *Equal Employment Opportunity Commission v. Catastrophe Management Solutions, Inc., case,* a Black employee had a job offer rescinded because they refused to cut their locs to conform to the company grooming policy.^32^ Furthermore, research shows that compared with White women, Black women spend more on hair care and products.^12,33,34^

Direct and indirect costs related to hair care or loss of job-related income impacts total take-home income and resources available to engage in health prevention, which subsequently affects long-term health outcomes. Income is a social determinant of health, and is implicated in health inequities in racial/ethnic minorities in the United States.

## Hair Discrimination and the Creating a Respectful and Open World for Natural Hair Act

The conversation, policies, and practices around hair discrimination are changing after local and national advocacy toward enactment of the Creating a Respectful and Open World for Natural Hair (CROWN) Act, which bans discrimination in hair texture and protective hairstyles in the workplace and schools. It was passed in the United States House of Representatives in 2020 and it is one step closer to becoming national law.^35^ Beginning with California, 7 states have passed the CROWN Act into law and 23 others have introduced this bill for consideration.^36^ Local and national advocacy surrounding this law will continue to provide an avenue for conversation and education toward ending hair discrimination.

## Conclusion

Actual (or fear of) hair discrimination can affect health behaviors in Black persons directly and indirectly. The relationships and paths from hair discrimination to health outcomes are complex, but discrimination is associated with numerous mental and physical health outcomes. Perceived hair discrimination or activities done to straighten hair to prevent hair discrimination or conform to discriminatory policies may place individuals at risk for disease (burns, hair loss, etc.) or risk for other risk factors (e.g., physical inactivity).

Although the experiences of hair discrimination addressed in this article have focused on natural hair and hair styles in persons of African descent, people with “non-natural hair” also face hair discrimination (prejudice toward Blacks who straighten their hair, prejudice toward certain hair colors, etc.). The purpose of this piece is to shed light on the long history and potential effects of hair discrimination on persons of African descent in the United States. In keeping with the tenets of equity, the authors are not stating that black natural hair is superior to other types, textures, or hair styles, but the purpose of this piece is to advocate for everyone's freedom to wear their hair in whatever manner they chose without the fear of prejudice or discrimination.

Passage of the CROWN Act brings us one step closer to this, but more work is needed to ensure that people should have the choice to style their hair however they choose, while still being able to engage in activities and behaviors that will lead to healthy lifestyles and positive health outcomes.

## Abbreviation Used

CROWN: Creating a Respectful and Open World for Natural Hair
